# The Impact of Pneumolysin on the Macrophage Response to *Streptococcus pneumoniae* is Strain-Dependent

**DOI:** 10.1371/journal.pone.0103625

**Published:** 2014-08-08

**Authors:** Richard M. Harvey, Catherine E. Hughes, Adrienne W. Paton, Claudia Trappetti, Rodney K. Tweten, James C. Paton

**Affiliations:** 1 Research Centre for Infectious Diseases, School of Molecular and Biomedical Science, University of Adelaide, Adelaide, Australia; 2 Department of Microbiology & Immunology, University of Oklahoma Health Sciences Center, Oklahoma City, Oklahoma, United States of America; Institut de Pharmacologie et de Biologie Structurale, France

## Abstract

*Streptococcus pneumoniae* is the world's leading cause of pneumonia, bacteremia, meningitis and otitis media. A major pneumococcal virulence factor is the cholesterol-dependent cytolysin, which has the defining property of forming pores in cholesterol-containing membranes. In recent times a clinically significant and internationally successful serotype 1 ST306 clone has been found to express a non-cytolytic variant of Ply (Ply_306_). However, while the pneumococcus is a naturally transformable organism, strains of the ST306 clonal group have to date been virtually impossible to transform, severely restricting efforts to understand the role of non-cytolytic Ply in the success of this clone. In this study isogenic Ply mutants were constructed in the D39 background and for the first time in the ST306 background (A0229467) to enable direct comparisons between Ply variants for their impact on the immune response in a macrophage-like cell line. Strains that expressed cytolytic Ply were found to induce a significant increase in IL-1β release from macrophage-like cells compared to the non-cytolytic and Ply-deficient strains in a background-independent manner, confirming the requirement for pore formation in the Ply-dependent activation of the NLRP3 inflammasome. However, cytolytic activity in the D39 background was found to induce increased expression of the genes encoding GM-CSF (*CSF2*), p19 subunit of IL-23 (*IL23A*) and IFNβ (*IFNB1*) compared to non-cytolytic and Ply-deficient D39 mutants, but had no effect in the A0229467 background. The impact of Ply on the immune response to the pneumococcus is highly dependent on the strain background, thus emphasising the importance of the interaction between specific virulence factors and other components of the genetic background of this organism.

## Introduction

*Streptococcus pneumoniae* (the pneumococcus) is responsible for almost 1 million deaths in children under 5 years of age annually, and is the leading cause of diseases such as pneumonia, bacteremia, meningitis and otitis media. A key virulence factor of the pneumococcus is the pore-forming toxin pneumolysin (Ply), which is a member of the family of cholesterol-dependent cytolysins and a potential target in future vaccine formulations [1]–[6]. Mutants lacking Ply have been shown to be attenuated in murine models of infection [7]. More specifically, Ply-deficient mutants have been characterised by reduced induction of pulmonary inflammation due to delayed cell recruitment into the lungs, particularly affecting neutrophil responses and the distribution of B and T lymphocytes in and around inflamed bronchioles [8], [9]. The defining property of Ply is its ability to form pores in cholesterol-containing membranes, causing potent induction of inflammation and tissue damage [7], [9]–[13]. However, Ply has a number of other properties including antibody-independent activation of the classical complement pathway and binding to toll-like receptor 4 (TLR4) [14]–[16]. Amongst a number of responses, pore formation activates the NLRP3 inflammasome, leading to Caspase-1 activation and subsequent processing and release of IL-1β from immune cells [17]. There is some evidence to suggest that Ply-induced pore formation is responsible for potassium efflux and lysosomal destabilization that are detected either directly by NLRP3 or via intermediary factors leading to inflammasome assembly and Caspase-1 activation [17]. Therefore, IL-1β release is indicative of Ply-dependent activation of the NLRP3 inflammasome [17].

Serotype 1 strains of *S. pneumoniae* are a significant cause of disease worldwide and are frequently responsible for outbreaks of IPD in otherwise healthy individuals [18]–[23]. In the last 20 years the ST306 serotype 1 clone has seen a dramatic expansion across the globe responsible for more than 80% of cases of serotype 1 disease in many jurisdictions [24]–[28]. Interestingly the ST306 clone expresses a non-cytolytic Ply variant that retains the ability to bind to cholesterol-containing membranes and oligomerise, but cannot form pores [25].

Previous work has also found that a hypervirulent serotype 1 strain 4496 of the clonal cluster 615 produced a Ply variant (Ply_4496_) with very low specific hemolytic activity, 1.6% of Ply_D39_ [29], [30]. However, when D39 was constructed to express Ply_4496_ the resultant mutant was similarly virulent to wild-type D39 in mice in terms of survival and was significantly more virulent than Ply-deficient D39 [29]. In addition, D39 constructed to express an engineered Ply toxoid PdT (D385N/C428G/W433F) that retains only 0.001% of Ply_D39_ hemolytic activity, was similar in virulence to D39 in terms of survival, but was significantly more virulent than the Ply-deficient strains [7]. The apparent importance of Ply to pathogenesis even in the absence of hemolytic activity highlights the lack of understanding of the relative importance of cytolytic activity to pneumococcal pathogenesis as a whole.

However, efforts to characterise the significance of this phenomenon have been frustrated by the genetically intractable nature of a number of serotype 1 strains such as those of the ST306 clonal group. While attempts to characterise low and non-cytolytic variants, such as Ply_4496_ and Ply_306_, respectively, have been performed [25], [29], [31], these studies have either relied on comparisons between different wild-type strains that express different Ply variants in the context of numerous other known and unknown strain-specific differences, or performed comparisons between isogenic strains, but in the context of an alternative strain background that is easily transformable. In both cases the significance of the interaction between the cytolytic activity of Ply and the clinically relevant strain background is not considered, representing a significant shortcoming in understanding the role of Ply in clinically important and internationally successful strains.

The current studies are the first to detail mutations made in a strain of the ST306 clonal group and the first to detail comparisons between the immune response to isogenic Ply mutants in either the D39 or ST306 (strain A0229467) backgrounds. Ply-deficient mutants (D39ΔPly & A0229467ΔPly) and mutants expressing engineered non-cytolytic derivatives that cannot bind to mammalian cell membranes, oligomerise or form pores (L460D [32]) were constructed in the D39 and A0229467 backgrounds. In this work the cytolytic activity of Ply was confirmed to be responsible for activation of the NLRP3 inflammasome in a strain-independent manner. However, more broadly the immune response of a macrophage-like cell line [33], [34] to different Ply variants was found to be highly dependent on the strain background and appeared to suggest that the naturally non-cytolytic strain had adapted to the lack of cytolytic Ply. In addition, this work highlights the significant interaction between specific virulence factors and other strain-specific components of the organism's genetic composition.

## Materials and Methods

### Ethics statement

This study was conducted in compliance with the Australian Code of Practice for the Care and Use of Animals for Scientific Purposes (7th Edition 2004) and the South Australian Animal Welfare Act 1985. All animal experiments were approved by the Animal Ethics Committee of the University of Adelaide. Ethics approval was obtained for molecular analyses of the A0229467 strain from the Central Australian Human Ethics Committee of the Northern Territory Department of Health.

### Strains and media

The virulent serotype 2 strain, D39 (NCTC 7466) was used in this study [35]. The serotype 1 blood isolate belonging to the ST306 clonal group (A0229467) was obtained from the Alice Springs Hospital. Serotyping and multi locus sequence typing were performed by the Public Health Microbiology Laboratory at Queensland Health Scientific Services. Opaque-phase variants of all strains were selected on Todd-Hewitt broth supplemented with 1% yeast extract (THY)-catalase plates [36], and these were used in all animal experiments. Before infection, the bacteria were grown in serum broth (SB) (nutrient broth [10 g/l peptone (Oxoid), 10 g/l Lab Lemco powder (Oxoid) and 5 g/l NaCl] and 10% [v/v] donor horse serum) to 1×10^8^ CFU/ml, and then diluted to the relevant challenge dose. For all other experiments, strains were grown at 37°C in a CO_2_-enriched atmosphere in a casein-based semisynthetic liquid medium (C+Y) [37] to 1×10^8^ CFU/ml. Bacteria were pelleted, washed and resuspended in PBS to 1×10^8^ CFU/ml.

### PCR

PCR reactions were performed using a Mastercycler Nexus thermal cycler (Eppendorf). Reactions were performed using the Phusion-Flash PCR system (Thermo Scientific). Overlap-extension PCR was carried out as previously described [38], [39].

### Transformation of pneumococci

For *S. pneumoniae* D39, preparation of competent cells and subsequent transformation was performed using the complete transformation medium (CTM) method [40], [41]. A0229467 was transformed by diluting an overnight culture in C+Y medium in a 1∶1 mixture of C+Y medium and Dulbecco's Modified Eagle's Medium (DMEM; Gibco, Grand Island, N.Y.) supplemented with 10% heat-inactivated fetal calf serum (FCS). After 2 h at 37°C, 50 ng/ml competence stimulating peptide 1 (CSP-1) was added, followed 15 min later by donor DNA (approximately 1 µg). After a further 2 h incubation at 37°C, transformation mixes were plated on blood agar supplemented with the appropriate antibiotics.

### Construction of Pneumolysin mutants

D39ΔPly was constructed by replacing the *ply* open reading frame with the gene encoding chloramphenicol acetyltransferase (*cml^R^*) and subsequent selection of chloramphenicol resistant recombinants. D39::Ply_306_ was constructed by replacing *cml^R^* in D39ΔPly with *ply_306_*, which was amplified from A0229467. D39::Ply_306_ recombinants were selected for the loss of chloramphenicol resistance as previously described [42]. D39::Ply_L460D_ was constructed by replacing *cml^R^* in D39ΔPly with the *ply*_L460D_ allele, which was generated by site-directed mutagenesis and overlap-extension PCR using primers RHPlyF_(5)_ (GGT GGT GCT TAT GCT TTG TCG), RHPlyR_(5)_ (GTG GGC AAT GAC AAA GGA TGT G), RHPlyL460DF (CCT GAG GAT AAT CAG TTG TTC C) and RHPlyL460DR (GGA ACA ACT GAT TAT CCT CAG G). D39::Ply_L460D_ recombinants were selected for loss of chloramphenicol resistance, as described above for D39::Ply_306_.

Ply mutants in the A0229467 background were constructed by replacing the *ply* ORF of a ST306 derivative with a *rpsL* mutation with the Janus cassette [43] (designated A0229467*rpsLply*::Janus). Insertion of the Janus cassette suppresses the *rpsL* Strep^R^ phenotype and confers kanamycin resistance. This construct was then transformed with genomic DNA from D39ΔPly and transformants were counter-selected by plating on BA+Strep. Genomic DNA was then extracted from a confirmed Strep^R^/Kan^S^ transformant that produced no Ply protein, as judged by western blotting and used to transform A0229467*rpsLply*::Janus. Deletion of the *ply* gene from one of the resultant second generation Strep^R^/Kan^S^ transformants (designated A0229467ΔPly) was confirmed by western blotting and DNA sequencing. This two-step procedure essentially eliminates co-transformation of other genetic markers from the donor DNA into the A0229467 background. An analogous procedure was used to replace the Janus cassette in A0229467*rpsLply*::Janus with the *ply* alleles from wild type D39 and D39::Ply_L460D_, generating A0229467::Ply_D39_ and A0229467::Ply_L460D_, respectively.

### Quantitative Western Blotting

Relative expression of Ply was determined by quantitative western blotting on whole cell lysates using the Odyssey Infrared Imaging System and associated software package (LI-COR Biosciences), as previously described [29].

### Hemolysis Assay

The hemolytic activity of pneumococcal lysates was determined as described previously [5], using PBS-washed human erythrocytes. Approximately 50 µg of total lysate supernatant protein was used for each sample in triplicate. The hemolytic activity was defined as the dilution factor at which 50% hemolysis was detected.

### Animal studies

Outbred 5- to 6-week old female CD1 (Swiss) mice were used in all challenge experiments. Mice were challenged intraperitoneally with 100 µl of bacterial suspension containing approximately 1×10^4^ CFU in SB. The challenge dose was confirmed retrospectively by serial dilution and plating of the inocula on blood agar. For survival experiments, mice were closely monitored for signs of illness over 14 days and were humanely euthanised when terminal infection was reached. Heart blood was taken from euthanised mice and plated on blood agar to confirm the presence of *S. pneumoniae*.

### Cell Culture and differentiation of THP-1 cells into a macrophage-like cell type

All tissue culture media and reagents were obtained from Gibco. THP-1 cells (ATCC TIB-202) were grown in 95% air-5% CO_2_ at 37°C in complete RPMI medium (RPMI with phenol red, supplemented with 10% FCS, 10 mM HEPES, 50 IU/ml penicillin and 50 µg/ml streptomycin). 10 cm dishes were seeded with 3.5×10^6^ THP-1 cells and differentiated by adding phorbol 12-myristate 13-acetate (PMA) to a final concentration of 10 ng/ml and incubating for three days at 37°C in 95% air-5% CO_2_. Differentiated cells attach to the plastic surface. Following the three day incubation cells were washed twice with complete RPMI and then rested at 37°C in 95% air-5% CO_2_ in complete RPMI for a further 3 days.

### Co-incubation of differentiated THP-1 cells with *S. pneumoniae*

Differentiated THP-1 cells were washed twice with PBS before adding 5 ml of RPMI without antibiotics, FCS, HEPES or phenol red. Bacteria (100 µl) were added to each dish at a 10∶1 (bacteria∶THP1 cells) ratio (100 µl PBS was added to each resting control) and incubated at 37°C in 95% air-5% CO_2_ for 6 h before RNA extraction, or 16 h if collecting supernatants for enzyme-linked immunosorbent assay (ELISA).

### ELISAs

Following 16 h co-incubation, the culture medium was collected and centrifuged at 16,000×*g* to pellet the bacteria. IL-1β in the resultant supernatants was quantified using the BD OptEIA Human IL-1β ELISA Kit II (BD Biosciences) and scanned on a SPECTROstar Omega microplate reader (BMG Labtech), according to the manufacturer's instructions.

### PCR Arrays

Following 6 h co-incubation, RNA was extracted from infected THP-1 cells using the PureLink RNA Mini Kit (Life Technologies), including on-column DNase digestion, according to the manufacture's instructions. Purified RNA was analysed on a LightCycler 480 II (Roche) by qRT-PCR using the RT^2^ Profiler PCR Array Human Innate and Adaptive Immune Responses kit (Qiagen), according to the manufacturer's instructions. Data were generated from quadruplicate samples.

### Statistical Analyses

The one-way ANOVA with the Tukey's multiple comparisons test was used to compare the mean survival times of mice, the mean relative Ply expression by quantitative Western Blot and the mean relative amount of IL-1β release by ELISA. The One-way ANOVA and Krustal-Wallis multiple comparisons test were performed using GraphPad Prism 6 for Mac. Statistical analyses for PCR Array data were performed according to the manufacturer's instructions. Changes in expression >2 fold and *P*<0.05 were considered statistically significant.

## Results

### Loss of the Ply cytolytic property increases mouse survival

**Figure 1:**
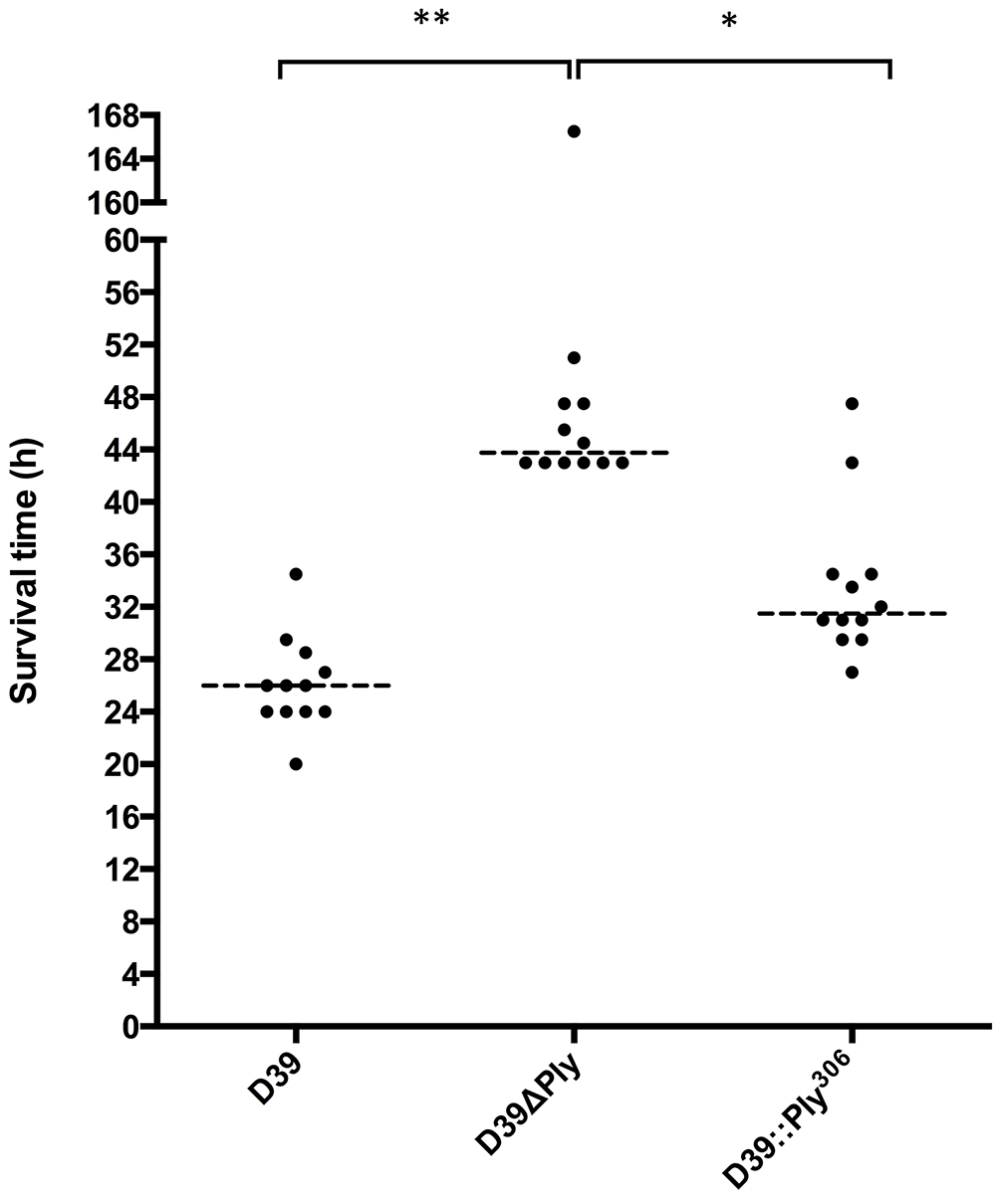
Survival of mice challenged with isogenic Ply mutants in D39. Survival times of groups of 12 CD1 mice challenged with 10^4^ CFU of the indicated strain via the intraperitoneal route of challenge. Statistical significance was calculated using the one-way ANOVA and Kruskal-Wallis and Dunn's multiple comparisons test (‘*’ *P*<0.05; ‘**’ *P*<0.01, ‘***’ *P*<0.001).

D39 was constructed to express the naturally occurring non-cytolytic Ply_306_ variant. The virulence of D39, D39ΔPly and D39::Ply_306_ was compared to determine whether the lack of cytolytic activity exhibited by Ply_306_ would lead to increased survival when expressed in D39. The survival times of groups of 12 mice challenged intraperitoneally with 10^4^ CFU of each strain D39, D39ΔPly or D39::Ply_306_ were compared (Fig. 1). The median survival time of mice challenged with D39ΔPly was significantly longer than both the wild type (*P*<0.001) and D39::Ply_306_ (*P*<0.05), but there was a much smaller but nevertheless statistically significant difference in median survival times between the wild type and D39::Ply_306_ (*P*>0.05). Thus, we confirm that while the cytolytic properties of Ply do contribute the virulence of wild type D39, the non-cytolytic properties appear to be much more important in this model.

### Induction of IL-1β release is dependent on the cytolytic activity of Ply but independent of the strain background

**Figure 2:**
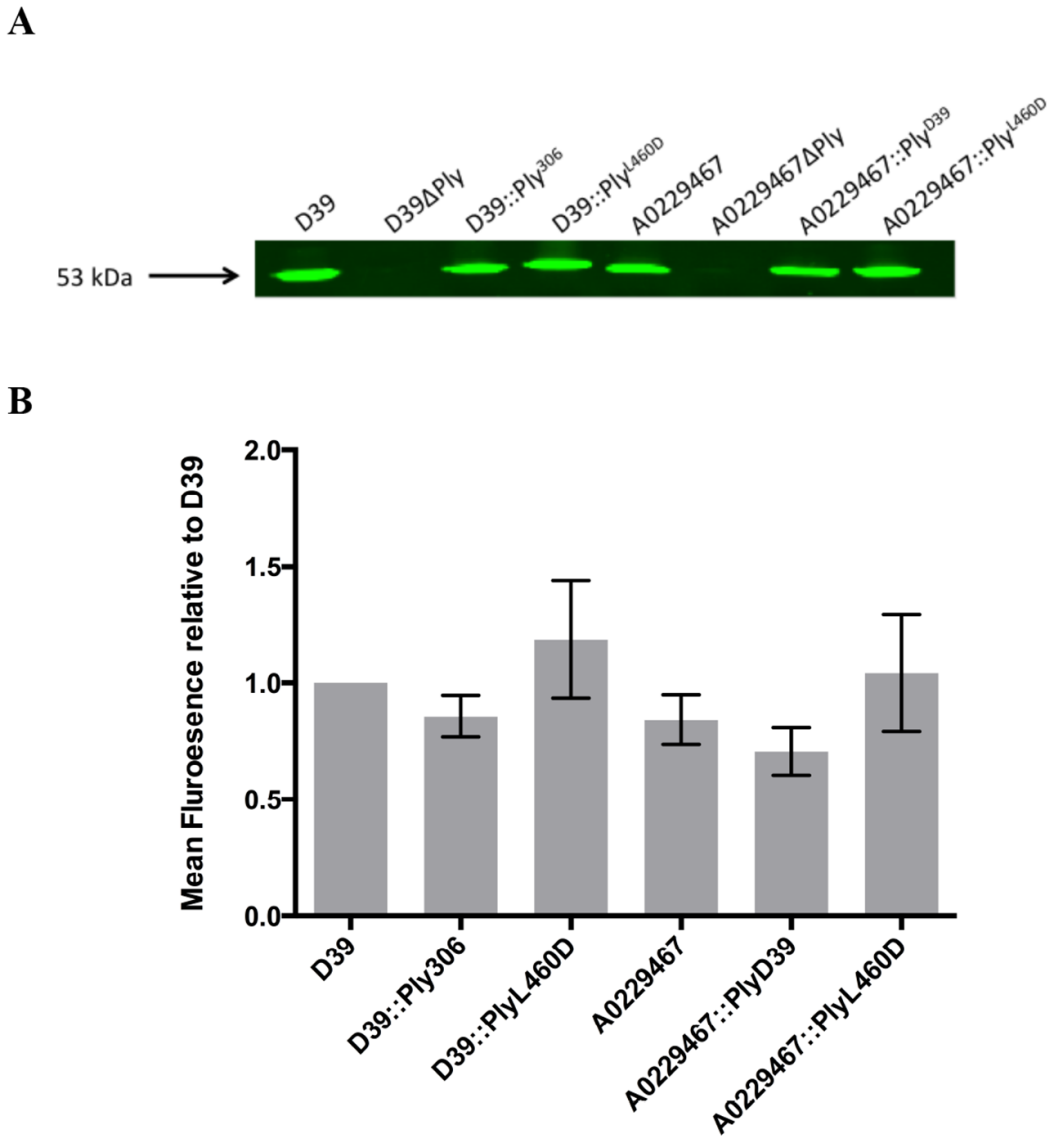
Ply expression. Ply expression analysis. Ply expression was compared between D39, D39ΔPly, D39::Ply306, D39::PlyL460D, A0229467, A0229467ΔPly, A0229467::PlyD39 and A0229467::PlyL460D by quantitative western blot using Ply-specific mouse antisera (A) The size of Ply (53 kDa) is indicated. The mean fluorescence units relative to D39 from biological triplicates are shown (B). Error bars indicate the SEM. Relative expression was compared between strains using the one-way ANOVA and Kruskal-Wallis and Dunn's multiple comparisons test, where *P*<0.05 would indicate statistical significance.

**Figure 3:**
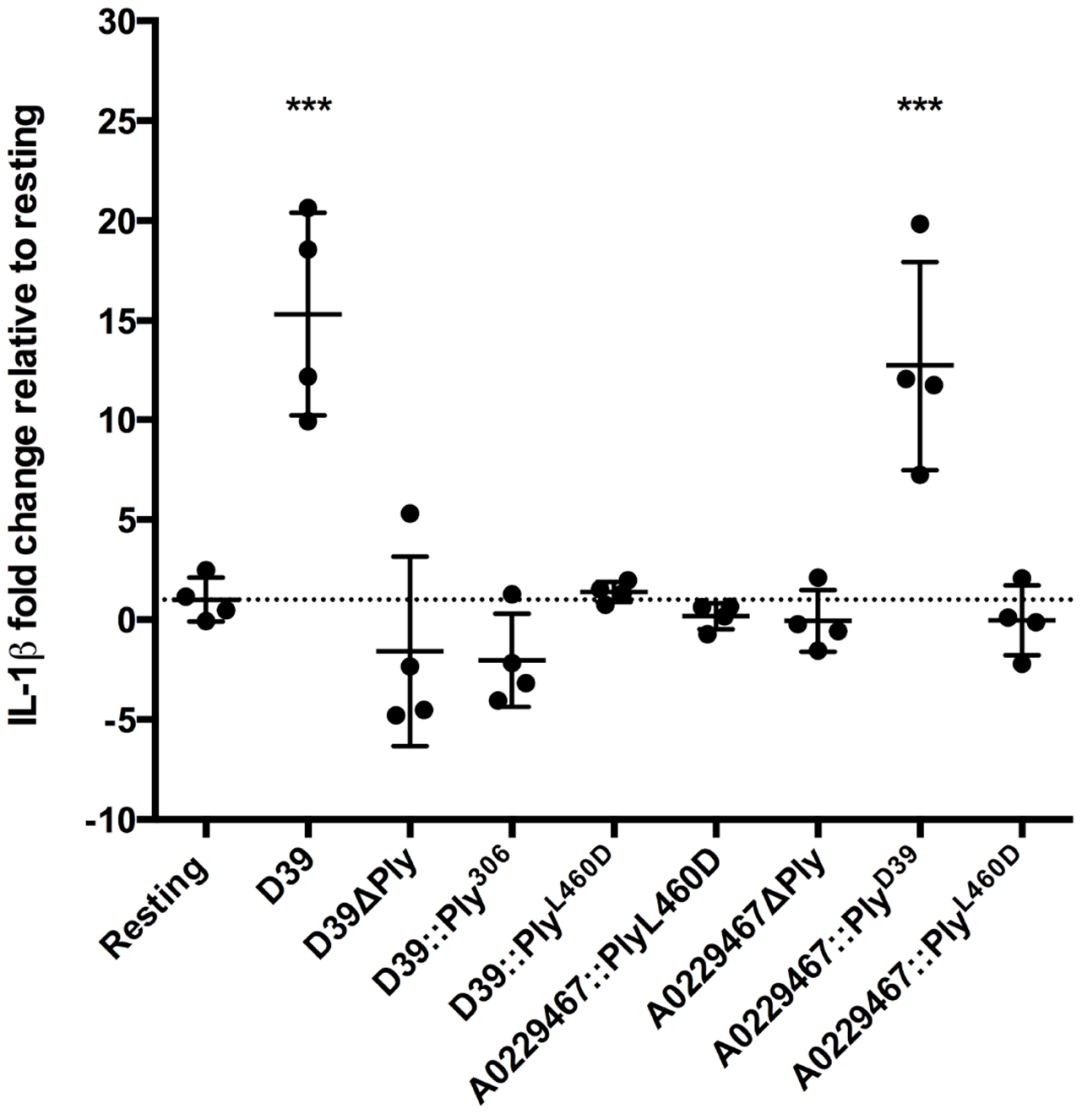
Ply-dependent induction of IL-1β release requires cytolytic activity. Differentiated THP-1 cells were co-incubated with bacteria at a 1∶10 ratio for 16 h. Supernatants were collected and IL-1β levels measured by ELISA. The mean quantity of IL-1β detected from resting samples is set at a relative value of 1 (dashed line), with all data points plotted as fold-change from this value. Error bars indicate the mean with standard deviation. Significant increases in IL-1β levels are highlighted, *** = *P*<0.001, n = 4.

Table 1 lists the isogenic Ply mutants that were used in this study and include mutants in both the D39 and A0229467 backgrounds. All 8 strains were cultured in C+Y broth, and aliquots were taken for western blot and hemolytic activity analyses. Quantitative western blotting of equal quantities of total lysate supernatant protein confirmed that there were no significant differences in the amounts of Ply protein produced by D39, D39::Ply_306_, D39::Ply_L460D_, A0229467, A0229467::Ply_D39_ and A0229467::Ply_L460D_ (Fig. 2B). Ply was not detected in either the D39ΔPly or A0229467ΔPly samples (Fig. 2A). Hemolysis assays were used to determine the specific hemolytic activity of equal quantities of total lysate supernatant protein of each strain. D39 and A0229467::Ply_D39_ exhibited hemolytic activity at approximately 3×10^3^ and 2×10^3^ HU/mg lysate supernatant protein, respectively (Table 1). Hemolytic activity was below the limit of detection (20 HU/mg lysate supernatant protein) for the remaining six strains, which confirms that the Ply_306_-expressing strains and Ply_L460D_-expressing strains are essentially non-hemolytic, as previously described [25], [32]. To confirm that lytic Ply (Ply_D39_) induces IL-1β release when expressed in either the D39 or A0229467 background, differentiated THP-1 cells were co-incubated for 16 h separately with all eight strains listed in Table 2. Approximately 15.3 and 12.7 fold more released IL-1β was detected in the presence of wild-type D39 and A0229467::Ply_D39_ compared to the resting control, respectively (Fig. 3). In contrast, there was no difference in the amount of IL-1β detected between the remaining six samples and the resting control. Therefore, Ply-dependent IL-1β release is associated with cytolytic activity of the toxin in a strain-independent manner, and confirms for the first time using isogenic strains that Ply-dependent activation of the NLRP3 inflammasome requires cytolytic activity in a strain-independent fashion.

**Table 1:** Hemolytic activity of the strains used in this study.

Strain	Specific Hemolytic Activity (HU/mg)^a^	Mutant Source (Ref)
D39	3×10^3^	-c
D39ΔPly	-b	[29]
D39::Ply_306_	-	This study
D39::Ply_L460D_	-	This study
A0229467	-	-c
A0229467ΔPly	-	This study
A0229467::Ply_D39_	2×10^3^	This study
A0229467::Ply_L460D_	-	This study

a Hemolytic units per mg of cell lysate supernatant protein.

b Hemolytic activity below the limit of detection of 20 HU/mg.

c Wild type strains.

**Table 2:** Ply-dependent changes in expression in the D39 background.

	D39 v D39ΔPly	D39 v Ply_306_	D39 v Ply_L460D_	D39ΔPly v Ply_L460D_
**Gene**				
*CSF2*	−6.94^**^	−9.15^**^	−13.99^**^	−2.01^**^
*IFNB1*	−6.18^**^	−5.35^*^	*ns*	*ns*
*IL23A*	−3.22^***^	−4.06^***^	−4.46^***^	*ns*
*CXCL10*	*ns*	2.15^*^	*ns*	*ns*
*IL8*	*ns*	−2.07^**^	**−2.41**	*ns*
*IRF7*	*ns*	−2.05^*^	*ns*	*ns*
*IL10*	*ns*	*ns*	−2.65^**^	*ns*
*IL1A*	*ns*	*ns*	−2.56^*^	*ns*

Significant changes in expression are ≥2 fold and *P*<0.05. (‘*’*P*<0.05; ‘**’ *P*<0.01; ‘***’ *P*<0.001).

*ns* denotes changes in expression that are not significant.

### Differential expression of immune genes in differentiated THP-1 cells is related to both the strain background and Ply variant

The ability to genetically manipulate a strain of the ST306 clone provided the opportunity to compare the immune response to isogenic Ply clones in that background and D39, and distinguish the contribution made by cytolytic activity to the immune response from that of the ability to bind to the eukaryotic cell surface. The immune response was characterised broadly and at the level of transcription between isogenic Ply mutants in the D39 and A0229467 backgrounds. The expression of 84 genes representing key pathways in the response against bacteria were analysed *in vitro* in PMA-activated THP-1 cells. Following PMA-driven differentiation into a macrophage-like cell type, these cells were incubated separately with each of the strains listed in Table 1 at a 1∶10 ratio. An additional unchallenged ‘resting’ control was included for each experiment. Significant changes in gene expression were deemed to be those with changes ≥2 fold and *P*<0.05. The impact of each strain on the viability of THP-1 cells following 6 h co-incubation was determined by trypan blue staining and no differences were found between any strain (data not shown).

For the comparisons in the D39 background, the genes encoding the p19 subunit of interleukin 23α (*IL23A*) and granulocyte macrophage colony-stimulating factor (GM-CSF [*CSF2*]) exhibited reduced expression in the presence of D39ΔPly, D39::Ply306 and D39::PlyL460D compared to wild-type D39, which suggests a link between the expression of these genes and cytolytic activity (Table 2). Interestingly, despite significantly reduced *CSF2* expression compared to the wild type, D39ΔPly was associated with significantly increased *CSF2* expression compared to D39::Ply_L460D_. In addition, both wild-type D39 and D39ΔPly were also associated with 16.17- and 2.33-fold (*P*<0.01) increased *CSF2* expression, respectively, compared to the resting group, whereas neither D39::Ply_306_ nor D39::Ply_L460D_, were associated with a change in *CSF2* expression. In contrast, *IL23A* expression was elevated 32.97 (D39), 10.23 (D39ΔPly) 8.12 (D39::Ply_306_) and 7.39 (D39::Ply_L460D_) fold (*P*<0.001) in the presence of each of the four strains compared to the resting group. Expression of the gene encoding interferon β1 (*IFNB1*) was reduced compared to the wild type in the presence of D39ΔPly and D39::Ply_306_, but not D39::Ply_L460D_ (Table 2). A small but significant reduction in the expression of the interleukin 8 (*IL8*) was detected in the presence of both D39::Ply_306_ and D39::Ply_L460D_ compared to the wild type. However, *IL8* expression was dramatically increased by 2206.18 (D39), 1133.71 (D39ΔPly), 1068.1 (D39::Ply_306_) and 915.76 (D39::Ply_L460D_) fold (*P*<0.01) in the presence of all four strains compared to the resting control, indicating that *IL8* expression is dramatically induced by the pneumococcus in a largely Ply-independent manner. Small but significant changes in expression were observed for chemokine (C-X-C motif) ligand 10 (*CXCL10*) and interferon regulatory factor 7 (*IRF7*) in the presence of D39::Ply_306_ compared to the wild type (Table 2). In addition, the expression of interleukin 10 (*IL10*) and interleukin 1α (*IL1A*) genes was reduced in the presence of D39::Ply_L460D_ compared to the wild type (Table 2).

In comparison to the differences observed between D39 strains expressing different Ply variants, very few changes in expression were detected between the A0229467 strains. A 2.10-fold (*P*<0.05) reduction in *IFNB1* expression was detected in the presence of A0229467ΔPly compared to the wild type. In contrast, A0229467::Ply_D39_ induced a 7.62-fold (*P*<0.05) increase in *IFNB1* expression compared to A0229467ΔPly. The expression of the gene encoding the CD8a molecule (*CD8A*) was reduced by 2.48-fold (*P*<0.05) in the presence of A0229467::Ply_L460D_ compared to A0229467ΔPly and the expression of the *CSF2* gene was reduced by 2.33-fold (*P*<0.01) in the presence of A0229467::Ply_D39_ compared to A0229467::Ply_L460D_. *CSF2* expression was increased by 5.99, 10.5 and 4.51 fold (*P*<0.05) in the presence of A0229467ΔPly, A0229467::Ply_D39_ and A0229467::Ply_L460D_ relative to the resting group, respectively.

### Ply-independent changes in gene expression

Table 3 lists the genes that exhibited changed expression between the resting groups and the challenge groups in a Ply-independent manner. In particular, the genes encoding complement component 3 (*C3*), the CD86 molecule (*CD86*), myeloperoxidase (*MPO*) and nucleotide-binding oligomerisation domain-containing protein 2 (*NOD2*) were significantly changed in the presence of D39, but not A0229467 (Table 3). In contrast to D39, 9 genes exhibited reduced expression in the presence of A0229467 compared to the resting group but were unaltered in the presence of D39 (Table 3). These included the genes encoding chemokine receptor 5 (*CCR5*), the CD4 molecule (*CD4*), CD40 ligand (*CD40LG*), CD11b (*ITGAM*), lymphocyte antigen 96 (*LY96*), nucleotide-oligomerisation domain containing protein 1 (*NOD1*) and toll-like receptor 1 (*TLR1*). Ply-independent changes in expression that were similar between the two strain backgrounds are listed in the supplementary section (File S1).

**Table 3:** Strain-dependent Ply-independent changes in expression relative to resting.

Gene	D39	D39ΔPly	D39::Ply_306_	D39::Ply_L460D_
*C3*	6.04^*^	4.01^*^	*ns*	3.54^*^
*CD86*	−2.51^*^	−2.49^*^	*ns*	−2.28^*^
*MPO*	2.16^*^	2.10^*^	2.63^*^	2.44^**^
*NOD2*	2.33^**^	2.87^**^	2.30^***^	2.51^**^

Gene	A0229467	A0229467ΔPly	A0229467::Ply_D39_	A0229467::Ply_L460D_
*CCR5*	−3.43^*^	−3.12^*^	−3.05^*^	−3.95^*^
*CD4*	−2.13^*^	*ns*	−3.05^*^	−3.08^*^
*CD40LG*	−3.25^**^	−3.23^**^	−3.39^**^	−2.97^*^
*ITGAM*	−2.80^**^	−2.42^*^	−3.28^**^	−2.43^**^
*LY96*	−2.64^**^	−2.61^**^	−2.33^**^	−2.08^**^
*NOD1*	−3.00^*^	−2.01^*^	−2.66^*^	−2.48^*^
*TLR1*	−2.37^***^	−2.50^***^	−3.77^***^	−3.75^***^

Significant changes in expression are ≥2 fold and *P*<0.05. (‘*’*P*<0.05; ‘**’ *P*<0.01; ‘***’ *P*<0.001).

*ns* denotes changes in expression that are not significant.

## Discussion

While the importance of Ply as a pneumococcal virulence factor has been known for decades, it is becoming increasingly clear that the role Ply plays in disease is more complex than originally thought. The apparent success of clonal types that express non-cytolytic variants of Ply suggest that cytolytic activity may not be an essential property in certain contexts. Furthermore, Ply variants with low (Ply_4496_ [29]) and absent (Ply_306_ [this study]) cytolytic activity suffer only small reductions in virulence compared to the fully cytolytic wild-type D39 [29]. Given that the Ply_306_-expressing D39 strain was shown to be significantly more virulent than Ply-deficient D39, it is possible to conclude that the non-cytolytic properties of Ply are essential for full virulence within the host. In previous work it has been suggested that strains expressing non-cytolytic Ply may have an advantage over cytolytic strains as they do not activate the NLRP3 inflammasome and may be less vulnerable to clearance from the host [17], [31]. One such study, which compared the ability of the cytolytic and non-cytolytic clinical isolates to trigger IL-1β release, suggested a role for the cytolytic activity of Ply in NLRP3 inflammasome activation [31]. However, given that the study was performed between different isolates it is difficult to resolve the contribution to NLRP3 inflammasome activation made by Ply from that of other strain-specific differences in genetic background. In contrast, the use of isogenic Ply mutants in the present study was able to confirm that only bacteria expressing the cytolytic Ply variant, PlyD39, were capable of triggering IL-1β release from cultured human macrophage-like cells, irrespective of the strain background.

Construction of isogenic Ply mutants in the D39 and A0229467 backgrounds provided an opportunity to broadly characterise the differences in the induced immune response associated with the different Ply variants in two backgrounds. This study examined the effects of strain background and Ply variant at the transcriptional level, which is one of the earliest measureable parameters of the cellular response to pathogens. It focuses on a select subset of genes known to be involved in the host immune response against bacteria [44]. Firstly the ability of Ply to bind to the cell surface was not associated with any consistent changes in gene expression in either background, as the Ply_306_ v Ply_L460D_ and Ply_D39_ v Ply_L460D_ comparisons showed. In the D39 background, the cytolytic activity of Ply induces increased expression of genes encoding pro-inflammatory cytokines, such as GM-CSF and IL-23α, as well as IFNβ1. *CSF2*, which encodes GM-CSF, remained either unchanged or was weakly up-regulated in the presence of D39 strains expressing no or non-cytolytic Ply, but was significantly induced upon expression of cytolytic Ply. GM-CSF stimulation of macrophages in culture has been shown to induce an activated/pro-inflammatory “M1” phenotype, leading to increased production of other pro-inflammatory cytokines upon exposure to bacterial components [45]. *In vivo* it is involved in recruitment of granulocytes, including neutrophils, and macrophages to sites of inflammation, as well as promoting survival and activation of these cell types within the tissue [45]. All D39 strains induced a significant increase in *IL23A* expression. However, the expression of cytolytic Ply in this background induced a much greater increase in *IL23A* expression than the other variants, which may at least in part be related to the induction of *CSF2*. IL-23 also induces expression of pro-inflammatory cytokines such as TNF and IL-6. It has been identified as an important factor in the maintenance and function of Th17 cells [46], [47], which have been shown to be key players in the host response against the pneumococcus [48], [49]. *IFNB1* expression was also only induced by D39 expressing cytolytic Ply, possibly due to the detection of bacterial nucleic acids in the cytoplasm of the THP-1 cells that may have entered via the Ply pore [50]. *CXCL10* was the only gene that exhibited increased expression in the presence of any one of the non-cytolytic variants versus the wild-type, which occurred in the presence of D39::Ply306 and may point to unknown properties of Ply.

Unlike D39, the A0229467 strains did not induce any consistent changes in gene expression that were associated with cytolytic activity. Interestingly, in contrast to the D39 strains, the overall effect of A0229467 strains seemed to be a general down-regulation of immune genes, independent of Ply expression. Whereas D39 induced up-regulation of a number of genes not induced by A0229467 (including *C3*, *MPO* and *NOD2*), A0229467-specific Ply-independent differential gene regulation was almost universally the down-regulation of genes, including *CCR5*, *CD4*, *ITGAM*, *NOD1* and *TLR1*. This might suggest a mechanism by which A0229467 can compensate for a lack of cytolytic activity by inducing a general suppression of the immune response in this cell line. A notable exception was the increased *CSF2* expression consistently associated with the A0229467 background. Contrary to the cytolytic Ply-dependent increase in expression seen in the D39 background, A0229467 appears to cause an up-regulation of *CSF2*, with only a slight increase in expression between cytolytic and non-cytolytic Ply-expressing strains. The dramatic increase in *IL8* expression observed for all strains is consistent with detection of bacterial cell components by pattern-recognition receptors and secondary stimulation by proinflammatory cytokines as IL-1 and TNF [51]–[54]. The implications of these Ply-independent changes in gene expression merit further investigation.

In summary, the ability to genetically manipulate A0229467 made it possible to perform the first direct comparisons between the immune response to isogenic Ply mutants in the naturally non-cytolytic A0229467 background. This work confirmed that the cytolytic activity of Ply is responsible for IL-1β release in a strain-independent manner. Furthermore, this work showed that the cytolytic activity of Ply was important for the immune response to the naturally cytolytic D39, but had very little impact on the response to the naturally non-cytolytic A0229467, suggesting that A0229467 has adapted to the absence of Ply cytolytic activity.

## Supporting Information

File S1 **Ply-independent changes in expression that were similar between the two strain backgrounds.**
(DOCX)
