# Supplementary material for: The Impact of Pneumolysin on the Macrophage Response to Streptococcus pneumoniae is Strain-Dependent
Source: PLoS One. 2014 Aug 8;9(8):e103625. doi: 10.1371/journal.pone.0103625 (PMC4126675; doi:10.1371/journal.pone.0103625)
Supplement: File S1 — Ply-independent changes in expression that were similar between the two strain backgrounds. (DOCX) [file pone.0103625.s001.docx]

**Table S1. Expression changes in challenged samples versus resting.**

|  | **D39** | **D39ΔPly** | **D39::Ply_306_** | **D39::Ply_L460D_** |
| --- | --- | --- | --- | --- |
| **Gene** |  |  |  |  |
| *CCL2* | **17.65^***^** | **18.62^**^** | **15.19^*^** | **16.01^**^** |
| *CCL5* | **4.04^***^** | **5.46^**^** | **4.21^***^** | **4.46^**^** |
| *CD14* | **-4.39^***^** | **-4.73^***^** | **-4.57^***^** | **-4.64^***^** |
|  |  |  |  |  |
| *CD40* | **27.93^**^** | **43.59^**^** | **33.71^***^** | **25.38^**^** |
| *CD80* | **30.59^**^** | **36.61^**^** | **45.99^**^** | **37.63^***^** |
| *ICAM1* | **13.90^**^** | **15.91^***^** | **13.99^***^** | **12.48^***^** |
|  |  |  |  |  |
| *IL1B* | **632.46^**^** | **696.67^**^** | **598.76^**^** | **480.65^***^** |
| *IL1R* | **-2.45^**^** | **-2.22^**^** | **-2.67^**^** | **-2.39^***^** |
| *I-6* | **684.94^**^** | **687.08^***^** | **556.73^**^** | **525.06^***^** |
|  |  |  |  |  |
| *MX1* | **4.69^**^** | **4.31^***^** | **4.37^***^** | **3.10^**^** |
| *NFKB* | **14.78^***^** | **15.06^***^** | **14.44^***^** | **13.06^***^** |
| *NFKBIA* | **18.84^**^** | **17.00^**^** | **19.82^**^** | **16.39^***^** |
|  |  |  |  |  |
| *SLC11A1* | **-4.14^*^** | **-5.21^*^** | **-4.96^*^** | **-4.40^*^** |
| *STAT4* | **23.74^**^** | **13.21^***^** | **15.30^**^** | **13.81^***^** |
| *TICAM1* | **5.75^*^** | **6.87^**^** | **6.84^**^** | **5.48^***^** |
|  |  |  |  |  |
| *TLR3* | **-4.26^***^** | **-4.81^***^** | **-2.88^**^** | **-3.95^***^** |
| *TLR7* | **-3.45^***^** | **-2.47^***^** | **-2.14^*^** | **-2.78^***^** |
| *TNF* | **38.01^***^** | **37.60^***^** | **36.86^**^** | **35.07^***^** |

Significant changes in expression are ≥ 2 fold and *P*<0.05. (‘*’*P*<0.05; ‘**’ *P*<0.01; ‘***’ *P*<0.001).

**Table S2: Expression changes in challenged samples versus resting.**

|  | **A0229467** | **A0229467ΔPly** | **A0229467::Ply_D39_** | **A0229467::Ply_L460D_** |
| --- | --- | --- | --- | --- |
| **Gene** |  |  |  |  |
| *CCL2* | **8.48^**^** | **7.46^*^** | **6.96^***^** | **8.98^***^** |
| *CCL5* | **2.55^*^** | **2.35^***^** | **2.48^*^** | **2.59^*^** |
| *CD14* | **-6.32^*^** | **-9.04^*^** | **-11.64^**^** | **-11.56^**^** |
|  |  |  |  |  |
| *CD40* | **40.95^**^** | **34.92^***^** | **30.30^***^** | **32.53^***^** |
| *CD80* | **15.53^**^** | **17.96^**^** | **11.71^**^** | **13.17^***^** |
| *ICAM1* | **16.36^***^** | **17.12^**^** | **12.43^***^** | **15.95^**^** |
|  |  |  |  |  |
| *IL1B* | **595.31^***^** | **623.83^***^** | **568.30^***^** | **411.71^***^** |
| *IL1R* | **-2.28^*^** | **-2.69^*^** | **-3.94^**^** | **-2.99^**^** |
| *IL6* | **1915.27^**^** | **1811.96^**^** | **1492.83^**^** | **1151.13^***^** |
|  |  |  |  |  |
| *MX1* | **2.61^**^** | **2.31^***^** | **2.95^*^** | **2.00^*^** |
| *NFKB1* | **19.30^***^** | **16.92^***^** | **16.04^***^** | **15.85^***^** |
| *NFKBIA* | **15.54^***^** | **14.43^***^** | **12.01^***^** | **12.01^***^** |
|  |  |  |  |  |
| *SLC11A1* | **-4.73^**^** | **-4.75^**^** | **-5.27^**^** | **-5.26^**^** |
| *STAT4* | **23.02^**^** | **23.14^***^** | **29.00^**^** | **25.25^***^** |
| *TICAM1* | **5.22^**^** | **4.16^*^** | **4.02^***^** | **4.56^***^** |
|  |  |  |  |  |
| *TLR3* | **-3.05^**^** | **-5.81^***^** | **-5.51^**^** | **-6.09^***^** |
| *TLR7* | **-2.69^***^** | **-3.14^***^** | **-4.77^***^** | **-3.26^***^** |
| *TNF* | **37.04^***^** | **32.86^***^** | **30.57^***^** | **26.65^***^** |

Significant changes in expression are ≥ 2 fold and *P*<0.05. (‘*’*P*<0.05; ‘**’ *P*<0.01; ‘***’ *P*<0.001).
